# Gender performances on social media: A comparative study of three top key opinion leaders in China

**DOI:** 10.3389/fpsyg.2022.1046887

**Published:** 2022-11-10

**Authors:** Ming Liu, Ruinan Zhao, Jieyun Feng

**Affiliations:** ^1^Department of Chinese and Bilingual Studies, The Hong Kong Polytechnic University, Hong Kong, Hong Kong SAR, China; ^2^Faculty of Humanities, The Hong Kong Polytechnic University, Hong Kong, Hong Kong SAR, China; ^3^School of International Studies, University of International Business and Economics, Beijing, China

**Keywords:** gender, social media, KOL, personality trait, speech act, addressing terms

## Abstract

This study gives a critical examination of the performances of three top key opinion leaders (KOLs) on social media in China to explore whether gender serves as an important social factor in their interaction with their followers. A critical analysis of their Weibo posts has revealed that they construct different gender identities through their preferential choices of personality traits, speech acts, and addressing terms. This can be explained in terms of their target consumers and the products they promote. It concludes that although gender can serve as an important factor in top KOLs’ performances on social media, it can be appropriated and exploited in varied ways to serve different communicative purposes.

## Gender and interaction

China has the world’s largest live-streaming market in terms of the scale of participation and revenue generation (Wang and Zhang, 2022). The COVID-19 crisis has further accelerated the growth of live-streaming even amid the economic downturn (Gai, 2022), as almost 9,000 live-streaming enterprises registered by the end of 2020, increasing by 360.8% compared to 2019 (iResearch, 2021). This has generated a revenue of 1.2 trillion yuan in 2020, reaching an annual increase rate of 197% (iResearch, 2021). The booming live-streaming market has given rise to an expanding team of key opinion leaders (KOLs) in China, who have attracted billions of active users on social media (Statista, 2022).

Since KOLs play important roles in diffusing information, setting agenda, and influencing others’ decision-making or behaviour (Luqiu et al., 2019), how KOLs interact with their followers have attracted growing research interest in the areas of public relations, communication, and marketing (Herring, 2003; Chambers, 2013; Wu and Lin, 2017; Zhang and Wu, 2018). Previous studies have identified a variety of linguistic and discursive strategies for KOLs to engage with their followers, such as emotional branding, speech acts, pronouns, addressing terms, emoji rhetoric, etc. (Ge and Gretzel, 2018; Zhang and Wu, 2018). Nevertheless, few studies have examined whether gender serves as an important social factor in KOLs’ performances on social media.

Previous studies on gendered interaction have identified different personality traits associated with males and females (Hayes, 2011; Lee and Lim, 2016). For example, females are perceived as warm, gentle, sensitive, emotional, and cautious, while males are associated with assertiveness, coarseness, toughness, aggressiveness, sternness, rationality, and self-confidence (Huddy and Terkildsen, 1993). Some other studies have also revealed different genders’ preferences for linguistic features (Newman et al., 2008). Newman et al. (2008) find that female language contains more personal pronouns, intensive verbs, and psychological references, while male language uses more swearing, articles, quantifiers, and spatial and long words. Leaper and Ayres (2007) also reveal that females are more likely to use affiliative speech acts, while males use more assertive speech acts.

Nevertheless, disputes remain over whether gender differences in language use will dissolve on social media. While the anonymity of social media might possibly lead to the hiding or invisibility of gender disparity, some previous studies have demonstrated that gender differences are still evident even when communication moves from offline to online (Herring and Kapidzic, 2015). Some others even argue that gender differences are not removed but might be magnified in online contexts (Guiller and Durndell, 2007; Cardo, 2020). Therefore, a critical examination of the gender performances of the top KOLs on social media can further shed light on the dynamic relations between gender and interaction on social media.

Besides, studies on gendered interaction “reached a deadlock partly because of the research question itself (looking for differences between women and men) and its presumptions (taking ‘gender’ for granted)” (Pavlidou, 2011, p. 411). This study takes a social constructionist view of gender as not “a constant, pre-given to interaction, but a product of it in which language plays a constitutive role” (Pavlidou, 2011, p. 411). It aims to examine the strategic use of language by top KOLs to achieve certain goals. Previous studies have demonstrated that both masculine and feminine traits can be appropriated by different genders in their strategic use of language (Fairclough, 1989; Wagner and Wodak, 2006; Holmes, 2008; Sung, 2013). Therefore, a critical examination of the gender performances by top KOLs can further demonstrate whether and to what extent gender emerges as a prominent strategy in their promotion.

## Contextualization of this study

A KOL is defined as “an individual who has a great amount of influence on the decision making, attitudes and behaviors of other people” (Casaló et al., 2020, p. 511) by virtue of their “knowledge, expertise, and authority over certain issues” (Casaló et al., 2020, p. 512). Most KOLs in China started from scratch, but created great commercial myths through their live-streaming practices. The live-streaming e-commerce has played a significant role in poverty alleviation, regional development, and economic and cultural transformations (Si, 2021).

This study focuses on three top KOLs, namely Li Jiaqi (Li), Viya, and Luo Yonghao (Luo). They were selected because of their influences as well as their distinctive gender performances. Li was once a makeup seller at a L’Oréal counter in Nanchang (a second-tier city in China). It is quite rare in China for a man selling cosmetics to women (Gai, 2022). However, Li’s persistence in selling beauty products finally won him the nickname “Lipstick King,” who has set the Guinness record of “the most lipstick applications to models in 30 s” during his live webcast (Guinness World Records, 2018). In a lipstick selling competition, Li sold 15,000 lipsticks in 5 min and beat the Alibaba founder Jack Ma (Teh, 2021).

Viya was the first female KOL of Taobao Live (Egg, 2020), and she earned the title of “Queen of Livestreaming.” Viya has occupied an “irreplaceable commercial value on the platform” (Si, 2021) since the day she initiated her live streaming. Before obtaining her reputation, Viya ran an apparel store and sold clothes in Guangzhou. She names herself “Dora Viya” (after Doraemon) because she is able to “sell anything.” The most impressive thing that she has sold was a rocket launch worthy of 40 million RMB in 2020. On Singles’ Day 2021, Li and Viya generated the most sales on Taobao (China’s biggest online shopping platform) at over 10 billion and 8 billion RMB, respectively.

Luo was the “first generation internet influencer” in China (Si, 2021, p. 88), whose teaching videos entitled “Lao Luo Quotations” became an Internet meme among young people in the 2000s. Known for his nonconformist and entrepreneurial spirits (Gao, 2017), he became the founder of the Chinese smartphone brand Smartisan Technology. His sharp remarks and several entrepreneurial transformations earned him a group of male followers who regard him as their spiritual leaders. But the company soon slipped into a debt turmoil for about 600 million RMB and he was put on the “deadbeat list” (credit blacklist). He had to engage in live-streaming practices to pay off his debts. Unlike other KOLs whose followers are largely females, Luo’s followers were primarily well-educated males who were attracted by Luo’s personal charisma (Mozur, 2015).

## Methodology

### Data collection

As a hybridized form of Twitter and Facebook, Sina Weibo is one of the most popular social media platforms in China. As a social networking platform, Weibo serves as a crucial channel for interpersonal communication (Han and Wang, 2015). It is also “an active agent in shaping public life and empowering grassroots advocacy” (Han, 2019, p. 380). This study collects original Weibo posts of Li Jiaqi (Li), Viya, and Luo Yonghao (Luo) from 1 April 1^st^ 2021 to 30 September 2021 (6 months). They are all “V-users” (verified users on Weibo) with millions of followers. Table 1 shows an overview of the Weibo accounts of the three KOLs. The three KOLs all have a strong sense of presence on Weibo, as their Weibo post entries were updated on a daily basis.

**Table 1:** Overview of the three key opinion leaders (KOLs’) Weibo accounts.

Name	Age	Gender	Followers (millions)	Total number	Average number (per day)
Li	30	Male	30.28	210	1.2
Viya	37	Female	18	278	1.5
Luo	50	Male	17.74	185	1.0

### Analytical framework and procedures

This study gives a critical discourse analysis (CDA) of gender construction in the Weibo posts of three top KOLs in Chinese mainland. CDA views language use as a social practice and underlines the significance of examining language use in its social contexts (Fairclough, 1989). It views language as socially shaped as well as socially shaping/constitutive and aims to examine the constitutive role of language use and the dynamic relations between language use, power, and ideology (Fairclough, 1989). Taking a social constructionist perspective, CDA views gender as socially constructed and aims to expose the (re)production, negotiation, and contestation of gender ideologies and power relations in a particular context (Wodak, 2003; Lazar, 2014). Due to the growing popularity of social media (Scardigno and Mininni, 2020; Ghaffari, 2022), a growing number of CDA studies have been conducted on the self-empowerment of females (Chang et al., 2016; Boling, 2019) and the construction of online gender identities (Peng, 2019; Ghaffari, 2022; Lane, 2022). For example, Ghaffari (2022) gave a critical examination of celebrities’ transgression of hegemonic gender stereotypes to generate an online hate speech. Peng (2019) also studied how a female Chinese KOL constructed a “feminized male ideal” identity to attract followers. These studies have revealed not only how gender identities can be negotiated or explored to meet a particular communication purpose but also the specific discursive strategies employed (Planchenault, 2010; Lane, 2022).

The constitutive effects of language can be identified in three aspects: (1) social knowledge; (2) social identities; and (3) social relations (Fairclough, 1992). While social knowledge is concerned with the experiential metafunction of language, social identities and social relations are related to the interpersonal metafunction of language (Halliday, 1994). The primary concern of this study is to examine the constructions of interpersonal relations by the three KOLs on their social media. It tries to address four research questions:

1. How do the three KOLs vary in their choice of linguistic features?
2. How do they construct relations between themselves and their followers?
3. What gender images do they construct for themselves on their social media?
4. How do they contribute to their respective communicative purposes?

In order to reveal the dynamic relations between gender and interaction, this study conducts a top-down analysis of these posts at three levels: (1) personality traits; (2) speech acts; and (3) addressing terms. Previous studies have demonstrated that men and women tend to display different personality traits. Men tend to be “determined,” “effective,” “fighting,” “forceful,” “confident,” “champion,” and “rational” (masculine traits), whereas women tend to be “caring,” “warm,” “compassionate,” “understanding,” “congenial,” “humble,” and “empathetic” (feminine traits) (Huddy and Terkildsen, 1993; Lee and Lim, 2016). This study starts with a critical examination of these posts to identify which personality traits each post communicates. Table 2 illustrates the personality traits identified. Since each post may communicate several personality traits simultaneously, this study identifies a maximum of two personality traits for each post for the convenience of analysis.

**Table 2:** Coding scheme for personality traits.

Types	Traits	Description	Illustrations
Masculine	Determined	Messages expressing KOLs are committed to achieving a goal, so that let no one to stop them.	继续勇往直前
			Keep moving
	Effective	Messages showing KOLs are skilled or able to do something well and achieve the intended results.	简单粗暴，先上福利
			Short and right to the point, benefits first
	Fighting	Messages conveying the will of refusing to give in and striving to compete.	困，是不存在的…
			Sleepy, no…
	Forceful	Messages expressing KOLs’ opinions very clearly and people are easily persuaded by them.	场面异常火爆
			The sales are booming
	Confident	Messages showing KOLs believe in their abilities to do things well.	爆款好物通通满足
			Satisfy your needs for best-sellers
	Champion	Messages conveying KOLs’ desire to be a winner or in the first place of a competition.	心动首发，各大新品抢先一步
			Attractive debut, all new products take the lead.
	Rational	Messages showing thoughts or decisions are based on reasons rather than emotions.	FILA 鞋跟上的这个饰件儿，应急的时候可以当吉他拨片用
			The accessory of FILA’s heel can be used as a guitar pick under emergency
Feminine	Caring	Messages expressing KOLs’ concerns for or kindness to others.	周日见，痘痘肌女生男生不要翘课哦 See you on Sunday, girls and boys with acne skin do not miss the class
	Warm	Messages showing KOLs are friendly or making others feel comfortable and relaxed.	一起收获快乐
			Harvest happiness together
	Compassionate	Messages conveying KOLs are feeling sympathy for people who are suffering or in bad situation.	帮助自强自立的乡村孩子圆梦未来
			Help the self-reliant rural children realize their dream of future
	Understanding	Messages showing KOLs have the knowledge of or familiarity with a particular thing.	这周的惊喜来咯
			Here’s this week’s surprise
	Congenial	Messages expressing KOLs have the same nature, disposition, or tastes with others.	放假啦，开心呀
			The vacation comes, happy
	Humble	Messages showing KOLs are not proud or arrogant but respectful.	很荣幸可以参加博鳌亚洲论坛2021年年会，让小李收获了不少新知识和新启发Very honoured to attend the 2021 Bo’ao Forum for Asia, little Li has learnt more new knowledge and got new inspirations
	Empathetic	Messages conveying KOLs are able to feel others’ emotions, thoughts or attitudes.	这…尽管没做错什么，但还是感到多少有点抱歉。
			This…although there’s no wrong-doing, I feel a little bit sorry.

Speech acts refer to the linguistic acts associated with the speaker’s utterance (Searle, 1965). Zhang and Wu (2018) make a distinction between self-oriented and other-oriented speech acts on social media. Self-oriented speech acts include three sub-categories: (1) “promoting self and others”; (2) “self-reporting mood”; and (3) “self-reporting moment and information.” Other-oriented speech acts include six categories: (1) “eliciting response”; (2) “directive”; (3) “greetings”; (4) “showing judgement and appreciation”; (5) “showing gratitude”; and (6) “congratulating.” Table 3 shows the illustrative examples for each speech act in the data. Although some posts communicate several speech acts at the same time, this study also identifies a maximum of two most prominent speech acts in a post for the convenience of analysis.

**Table 3:** Illustrative examples for speech acts.

Types	Speech acts	Description	Examples
Self-oriented	Promoting self and others	Messages promoting the KOLs’ own or others’ products.	超多品质好物买到嗨
			So many high-quality goods to have a happy shopping
	Self-reporting mood	Messages expressing emotions or inner feelings.	今天超级开心
			Today I’m super happy
	Self-reporting moment and information	Messages sharing small life stories or personal information.	打卡
			Check in
Other-oriented	Eliciting response	Messages eliciting responses from followers or the addressed.	你们想听什么课?
			What lessons do want to take?
	Directive	Messages expressing a request to others, directly or indirectly.	速来围观
			Come and join
	Greetings	Messages conveying daily, seasonal, birthday or festive greetings.	欢迎大家来到我的狂欢节
			Welcome to my Shopping Spree
	Showing judgement and appreciation	Messages expressing KOLs’ beliefs or evaluations of people and things.	用一句话说就是真的太划算了吧!
			One sentence to describe it “what a deal”
	Showing gratitude	Messages expressing thankfulness or gratitude to others.	谢谢你们的支持和信任
			Thank you for your support and trust
	Congratulating	Messages congratulating others for their outstanding performance or work.	恭喜@蔡少芬 刷新李佳琦仓库扫货新纪录
			Congratulations @Cai Shaofen Break the shopping record of Li Jiaqi’s stock

They are followed by a close analysis of the self- and other-addressing terms used by the three KOLs on their social media. All the pronouns and nominal forms used to address themselves and others are examined (Fairclough, 1992). They are examined in terms of gender attributes and power relations (Yang and Wang, 2022). Finally, their choices of personality traits, speech acts, and addressing terms are further interpreted in terms of their contributions to the construction of different gender images and social relations and explained in terms of their communicative purposes.

The coding of personality traits and speech acts was performed by the first and second authors. The two authors coded the data independently after the initial training and discussion of the coding criteria and procedure. The discrepancies in some results were resolved through further discussion. The inter-rater reliability was measured with Cohen’s Kappa. The average percent agreement of all coded categories was greater than 90%, and the Cohen’s Kappa was 0.91, indicating a strong agreement between the two coders (Cohen, 1960; Hallgren, 2012).

## Data analysis and discussion

### Analysis of personality traits

**Figure 1:**
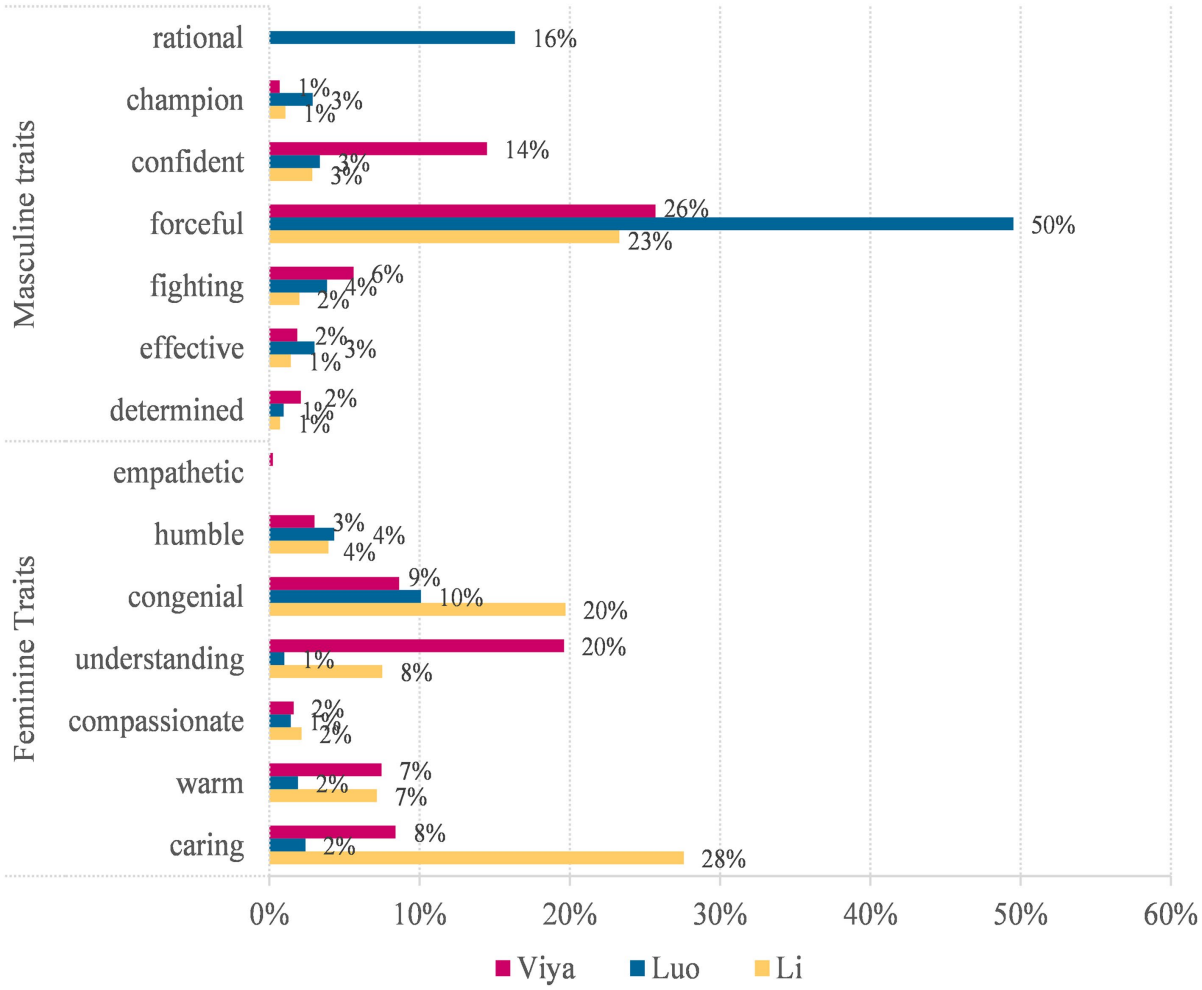
Distribution of personality traits among key opinion leaders (KOLs).

Table 4 and Figure 1 show the distribution of masculine and feminine personality traits on the Weibo posts of the three KOLs. Overall, Li shows a preference for feminine (190, 69%) over masculine traits (89, 31%), while Luo shows a preference for masculine (165, 80%) over feminine traits (43, 20%). In between is Viya, who shows a relatively balanced choice of masculine (216, 51%) and feminine traits (212, 49%). The findings are interesting in view of the fact that Viya is a female, while Li is a male.

**Table 4:** Frequencies of personality traits.

		Li		Luo		Viya	
Types	Traits	Freq	%	Freq	%	Freq	%
Feminine	Caring	77	28	5	2	36	8
	Warm	20	7	4	2	32	7
	Compassionate	6	2	3	1	7	2
	Understanding	21	8	1	1	84	20
	Congenial	55	20	21	10	37	9
	Humble	11	4	9	4	15	3
	Empathetic	0	0	0	0	1	0
	Sub-total	190	69	43	20	212	49
Masculine	Determined	2	1	2	1	9	2
	Effective	4	1	5	3	8	2
	Fighting	7	2	8	4	24	6
	Forceful	65	23	103	50	110	26
	Confident	8	3	7	3	62	14
	Champion	3	1	6	3	3	1
	Rational	0	0	34	16	0	0
	Sub-total	89	31	165	80	216	51
Total		279	100	208	100	428	100

They also show distinct differences in their preferences for specific personality traits. Luo is noted for his highest preferences for the masculine traits of “forceful” (50%) and “rational” (16%). Li shows the highest preferences for the feminine traits of “caring” (28%) and “congenial” (20%) and the masculine trait of “forceful” (23%). Viya shows the highest preferences for the feminine trait of “understanding” (20%) and the masculine traits of “forceful” (26%) and “confident” (14%). It shows that the personality trait “forceful” is valued by all three KOLs, which can be attributed to the primary function of their Weibo posts, i.e., to promote their products and engage the followers to buy their products (see Example 1). However, it is much more highlighted in Luo’s Weibo posts than in the other two KOLs’, suggesting that Luo prefers to deliver information about his products in a more straightforward way than the other two KOLs. This contributes to the construction of his “*zhinan*” (straight male) image.

Besides, Luo shows a “rational” trait by questioning, challenging or criticizing some social phenomenon or popular ideas (see Example 2). He disaligns himself with the public and the popular ideas, thus further consolidating his image of being the “spiritual leader” of some youngsters or middle-aged men. In other words, Luo does two things on his Weibo posts: promoting the products and constructing a critical image for himself. More often than not, they are achieved on separate posts. Examples are as follows:

(1) Forceful:

全场平均5折!全场平均5折!全场平均5折! (07/05/2021, Luo).

Average 50% off for all items! Average 50% off for all items! Average 50% off for all items!

(2) Rational:

虽然 Chord 是人类做过的最丑的解码器，但音质确实好…你看，仅从商业角度来说，电子产品的设计没那么重要。(24/09/2021, Luo).

Although Chord is the ugliest decoder made by humans, its sound quality is indeed good…You see, just considering business, the design of electronic products is not that important.

By contrast, although Li also promotes his products by underlining the “forceful” trait, he shows equal, if not more, preferences for the “congenial” and “caring” traits (see Examples 3 and 4). In other words, Li shows alignment with the followers by constructing himself as a “*guimi*” (i.e., ladybro) of his overwhelming female followers. Usually, they are achieved on the same Weibo post. That means Li promotes his products through constructing himself as a congenial and understanding friend of his followers. As Example 5 shows, Li shows a “forceful” trait by announcing the news on the one hand and a “caring” trait by asking the followers whether they are ready or not on the other hand. In example 6, Li shows a “forceful” trait by announcing the news and a “congenial” trait by showing he and his followers share the same hobby at the same time.

(3) Caring:

夏天了，有没有想吃的零食呀特别是那种私藏零食或者家乡美食，给我抄抄作业呀 (30/06/2021, Li).

Summer is coming, have any snacks wanna try? Especially those personal treasures or hometown specialties, let me copy your homework.

(4) Congenial:

我的宵夜和最近困困的Never胖死我算了 (28/05/2021, Li).

My night meal and the recent sleepy Never will become too fat

(5) Forceful + Caring:

正式官宣!#所有女生的offer#倒计时2天咯。准备好了吗，9月27号12:00见!(25/09/2021, Li).

Official announcement! # All Girls’ Offer # Countdown to 2 days. Ready? See you on 12:00 Sep. 27^th^!

(6) Forceful + Congenial:

今晚10点，他来咯!我就差报身份证号啦!(06/09/2021, Li).

Tonight 10 O’clock, he’s coming! Only the ID number has not been disclosed!

Viya is different in that she promotes her products by constructing herself as an expert in the field. On the one hand, she highlights that she understands what the followers need; on the other hand, she underlines her confidence in the quality and prices of her products. This is often achieved on the same posts (see Examples 7 and 8). In other words, she promotes her products because she believes that it is the best choice for the followers. The image she constructs for herself is a professional image rather than a gendered image. Examples are as follows:

(7) Forceful + understanding:

今晚#薇娅直播间# 99爆品日!vivo、雅诗兰黛新品首发!玉泽、雅萌ACE、OLAY超红瓶、笔记本电脑、百威啤酒…全家人都需要的好物就在今晚! (09/09/2021, Viya).

Tonight #Viya’s Live Room# 99 Top-sellers’ Day! Vivo, Estée Lauder debut! Dr. Yu, Yaman ACE, Olay Regenerist, laptop, Budweiser…Handy products for the whole family are here tonight!

(8) Forceful + Confident:

今晚 #薇娅直播间# 零食节开吃啦!文和友、好利来、星巴克、自嗨锅、马迭尔冰棍、好利来甜品、北海牧场新品酸奶..0.60 + 无限回购的爆款零食，不来亏炸! (14/07/2021, Viya).

Tonight #Viya’s Live Room# Snack’s Day Start eating! Wenheyou, Hollyland, Starbucks, self-heating hotpot, Modern ice cream, Hollyland dessert, Beihai Ranch new arrival yogurt…60 + repurchase top-selling snacks, you’ll suffer great losses if not come!

They highlight different personality traits to construct different gender images for themselves on their Weibo posts. Luo prefers to construct a “straight male” image for himself, Li a “ladybro” image while Viya a “professional” image. This can be attributed to the types of products they promote and the target followers of their Weibo posts.

### Analysis of speech acts

**Figure 2:**
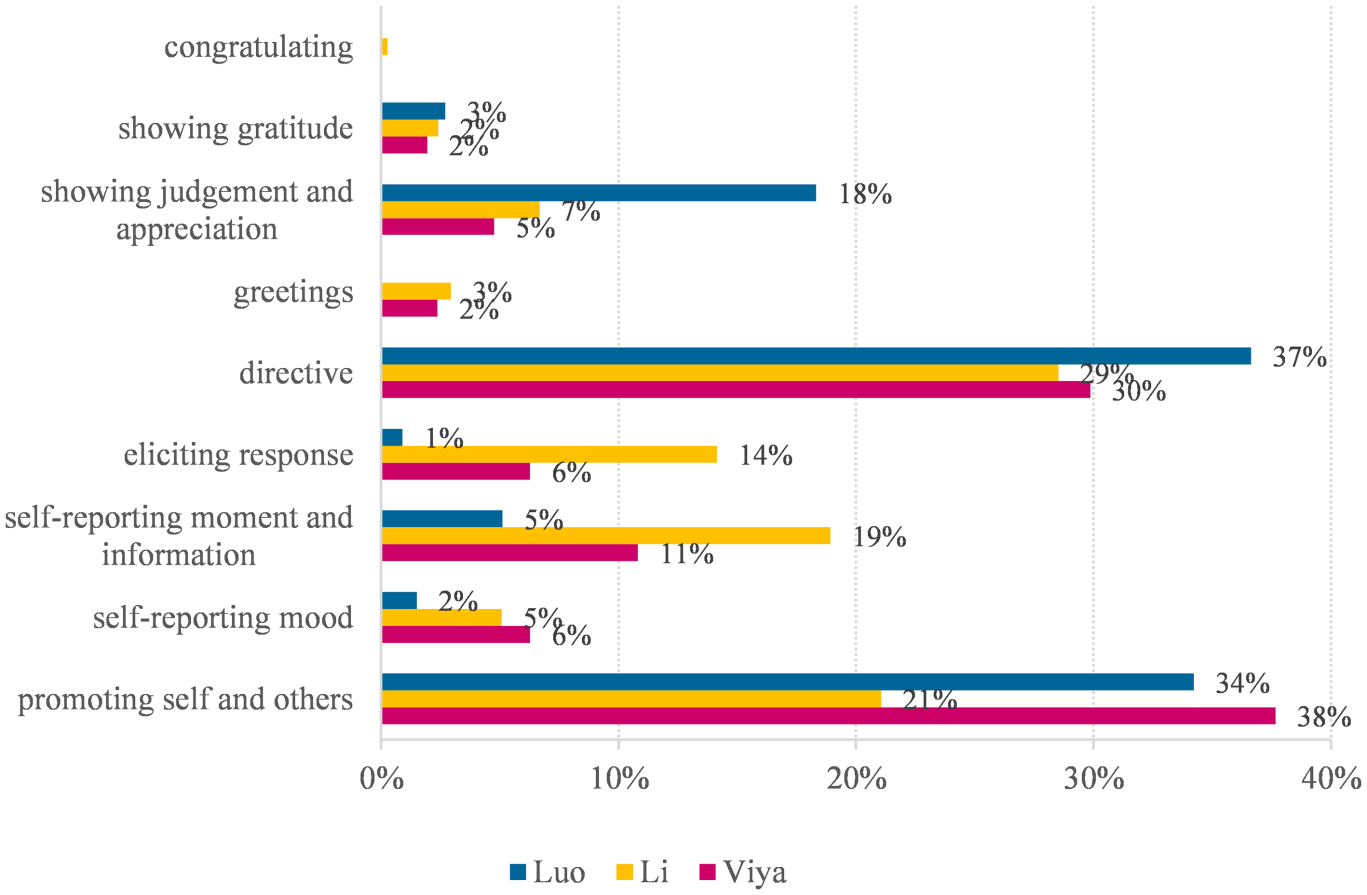
Distribution of speech acts among KOLs.

Table 5 and Figure 2 show the distribution of self-oriented and other-oriented speech acts on the three KOLs’ Weibo posts. Overall, Viya shows a preference for self-oriented speech acts over other-oriented speech acts (55% vs. 45%), while Luo (41% vs. 59%) and Li (45% vs. 55%) show the opposite. In other words, Viya is more self-oriented, while Luo and Li are more other-oriented. This is consistent with the above finding that Viya is more concerned about constructing a professional image, while Li and Luo prefer to construct a gender image.

**Table 5:** Frequencies of speech acts.

		Li		Luo		Viya	
Types	Speech acts	Freq	%	Freq	%	Freq	%
Self-oriented	Promoting self and others	79	21	114	34	174	38
	Self-reporting mood	19	5	5	2	29	6
	Self-reporting moment and information	71	19	17	5	50	11
	Sub-total	169	45	136	41	253	55
Other-oriented	Eliciting response	53	14	3	1	29	6
	Directive	107	29	122	37	138	30
	Greetings	11	3	2	0	11	2
	showing judgement and appreciation	25	7	61	18	22	5
	showing gratitude	9	2	9	3	9	2
	congratulating	1	0	0	0	0	0
	Sub-total	206	55	197	59	209	45
	Total	375	100	333	100	462	100

Besides, the three KOLs also show distinct differences in the choice of specific speech acts. Among all speech acts, “directive” and “promoting self and others” are most frequently used by three KOLs. “Promoting self and others” is related to the primary function of their Weibo posts, i.e., to promote their products (see Example 10). “Directive” is used to ask the followers to buy their products (see Example 9).

(9) Directive:

你没看错，今天周五，全场平均五折，现在就来我直播间抢购!(23/04/2021, Luo).

You did not misread it, today’s Friday, average 50% off for all items, now come and snap up in my live room!

(10) Promoting self and others:

今晚#薇娅零食节# 来啦!文和友、肯德基、必胜客、星巴克..超多美食让爱爱爱不完!(17/04/2021, Viya).

Tonight #Viya’s Snack Day# Comes! Wenheyou, KFC, Pizza Hut, Starbucks…too many snacks to enjoy them all!

As Figure 2 shows, Luo is noted for his highest preference for directive. However, Viya is noted for her highest preference for “promoting self and others.” This suggests that Luo is more straightforward in giving directions, while Viya is more concerned about promotion. Therefore, Luo tends to be more face-threatening than Viya, which is consistent with his “straight man” image.

Among the three KOLs, Li shows the lowest preference for “promoting self and others,” which suggests that he values soft sell over hard sell. This can be further seen from Li’s highest preferences for “self-reporting moment and information” and “eliciting response.” They suggest that Li prefers to interact with the followers by sharing his private life and engaging the public. Hereby he constructs himself as a congenial and caring ladybro of his followers. Examples are as follows:

(11) Self-reporting moment and information:

到上海了，开始工作用着贵妇面膜回回血我困，我感觉今晚直播会胡言乱语 (22/09/2021, Li).

Arrive in Shanghai, start working, refresh myself with a lady’s facemask, I’m sleepy, feeling that I’ll wander in my talk tonight.

(12) Eliciting Response:

今晚刷酸小课堂哦 你们有什么问题要问吗#李佳琦直播##李佳琦小课堂# (08/08/2021, Li).

Tonight peeling class Do you have any questions # Li Jiaqi’s Live Room# #Li Jiaqi’s small class#.

By contrast, Luo shows his highest preference for “showing judgement and appreciation.” That means that Luo is never afraid of expressing his own opinions towards some social phenomena or some things, thus constructing himself a key opinion leader or spiritual leader of youngsters and middle-aged men, as in the following:

(13) Showing judgement and appreciation:

卖了一年多的货，有些同事已经渐渐不说人话了，比如某个选品现货少，不够卖，说成“库存深度不足” 不说人话的吸引力究竟在哪里呢?(26/05/2021, Luo).

Have been selling for over 1 year, some colleagues have gradually not been able to speak like a normal man, for example “some product is in short supply, insufficient for sale,” they’ll say “stock in deep short supply” what’s the appeal in saying unlike human?

### Analysis of addressing terms

Table 6 shows the frequencies of addressing terms they use to refer to themselves and others. Among these addressing terms, personal pronouns are the most frequently used. Some personal pronouns are used for themselves, such as 我(I/me), and我们(we/us), whereas some personal pronouns are used to address others, including 你(you), and你们(you plural). They are most frequently used in Viya (196, 73%), followed in turn by Luo (109, 64%) and Li (130, 55%). It suggests that Viya prefers to use personal pronouns to address each other. Besides, among these personal pronouns (see Table 7), Viya (120, 40%) shows more preferences for second-person pronouns, followed in turn by Li (73, 29%) and Luo (38, 18%). In other words, Li shows the lowest preference for addressing the followers in a normal way, while Viya tends to address the followers in a normal way.

**Table 6:** Frequencies of KOLs’ addressing terms.

KOL	Personal pronouns		Nominal addressing terms	
	Raw	Standardized	Raw	Standardized
Li	130	20,240	107	16,659
Viya	196	17,296	72	6,354
Luo	109	7,478	60	4,116

**Table 7:** Addressing terms of KOLs.

KOL	Others	Self
Li	你们(*you* plural, 37), 你(*you* singular, 36),(所有)女生(*all girls*, 22),(所有)女生男生(*all girls and boys*, 4), 大家(*everybody*, 19), MM们(*beauties*, 4), 美眉们(*beauties*, 1), 宝妈宝爸们(*moms and daddies*, 3), 小伙伴们(*little friends*, 3),课代表们(*representatives*, 1),中奖绝缘体们(*jackpot insulator*, 1)	我 (*I*, 57), 我们(*we*, 17), 佳琦(*Jiaqi,* 15), 小李(*little Li*, 8), 小李老师(*Teacher Xiao Li*, 5), 李老头(*Old Li*, 2), 李时髦(*fashionable Li*, 2), 古风小李(*antique Xiao Li*, 2), 古装小李(*little Li in ancient costume*, 2), 李姓经纪人(*agent Li*, 2), 首席发现官(*chief discovery officer*, 2), 国风小李(*Chinese-style Xiao Li*, 1), 老板(*boss*, 1), 李店长(*shop manager Li*, 1), 阿里巴巴首席金牌助威官(*Alibaba chief gold cheering officer*, 1), 北境神推手(*marvelous recommender in the north*, 1), “小李”飞刀(*Dagger Li*, 2), 魔鬼李佳琦(*devil Li Jiaqi*,1), 李严格 (*serious Li*, 1), 全球首席偏爱官(*global chief preference officer*, 1)
Viya	你(*you* singular, 88), 你们(*you* plural, 32), 大家(*everybody*, 28), 小伙伴们(*little friends*, 9), 薇娅的女人骑士们(*Viya’s women and knights*, 4), 盆友们(*friends*, 1), 宝妈们(*moms*, 1), 时尚达人们(*fashion icons*, 1), 体验官(们)(*experience officers*, 8)	我(*I*, 76), 我们(*we*, 30), 薇娅(*Viya*, 4), 主播(*live streamer*, 3), 大掌柜(*big boss*, 3), 发起人(*initiator*, 2), 古风娅(*antique Viya*, 1), 薇娅(*Viya*, 1), 红色推荐官(*red recommender*, 1), 品鉴团团长(*commander of the review team*, 1), 品牌星鉴官(*senior brand review officer*, 1), 天猫小黑盒 “开新官” (*TMALL little black box new product release officer*, 1), 杭州市反诈骗宣传大使(*Hangzhou anti-fraud ambassador*, 1), 微博电商号首席好物优选官(*chief selection officer of Weibo e-commerce good products*, 1)
Luo	你(*you* singular, 33), 你们(*you* plural, 5), 大家(*everybody*, 19), 朋友(们) (*friends*, 10), 各位(*everyone*, 1), 网友们(*netizens*, 3), 小伙子(*young fellow*, 1), (XX, 7), 贱人们(*bitches*, 2), 神经病(*psycho*, 1), 真老赖(*real deadbeat*, 1), 流氓(*rogue*, 1),土豪大哥洋财主大姐(*rich redneck big brother foreign rich man and big sister*, 1), 各路善长仁翁(*all generous benefactors*, 1)	我(*I*, 71), 我们(*we*, 40), 老罗(*old Luo*, 6),罗永浩(*Luo Yonghao*, 4), 罗老师(*Teacher Luo*, 1), 这个星球上最优秀的软件产品经理(*the most excellent product manager in the world*, 1), 我们这些北方人(*we northerners*, 1)

In their choice of nominal addressing terms, Luo prefers to highlight his expertise and achievements in his field. In other words, he prefers to brag about his prestige and expertise, as can be seen from such expressions as “老罗” (*Old Luo*, 6), “罗老师” (*Teacher Luo*, 1), and “这个星球上最优秀的软件产品经理” (*the most excellent product manager in the world*, 1). Viya prefers to highlight her authority in her profession, such as “红色推荐官” (*Red recommender*, 1), “品鉴团团长” (*Commander of the review team*, 1), “品牌星鉴官” (*Senior brand review officer*, 1), “天猫小黑盒‘开新官’” (*TMALL little black box new product release officer*, 1), “杭州市反诈骗宣传大使” (*Hangzhou anti-fraud ambassador*, 1), and “微博电商号首席好物优选官” (*Chief selection officer of Weibo e-commerce good products*, 1). She prefers to highlight her title as an official to construct her social esteem in her profession. In other words, she aligns with the established social recognition. Therefore, both Luo and Viya construct themselves as having a higher social status than the followers. However, Luo prefers to use self-designated labels, while Viya prefers to highlight social recognition. By contrast, Li prefers to locate himself at a lower power position to his followers by addressing himself as “佳琦” (*Jiaqi*, 15), “小李” (*Little Li*, 8), “小李老师” (*Teacher Xiao Li*, 5), “李老头” (*Old Li*, 2), “李时髦” (*fashionable Li*, 2), “古风小李” (*antique Xiao Li*, 2), “古装小李” (*little Li in ancient costume*, 2), “李姓经纪人” (*agent Li*, 2), “国风小李” (*Chinese-style Xiao Li*, 1). Through using self-depreciating terms, nicknames, and first names, Li seeks to construct solidarity with his followers.

Their different preferences can also be seen from the nominal terms they used to address others. Luo uses not only some ironic terms but also some abusive terms to address others, including “XX” (7), “贱人们” (*bitches*, 2), “神经病” (*psycho*, 1), “真老赖” (*real deadbeat*, 1), “流氓” (*rogue*, 1), “土豪大哥洋财主大姐” (*rich redneck big brother foreign rich man and big sister*, 1), “各路善长仁翁” (*all generous benefactors*, 1). In other words, he prefers to attack or satirize some people, thus constructing an aggressive or cynical image for himself. However, Viya prefers to use some nicknames or internet buzzwords to address others, such as “小伙伴们” (*little friends*, 9), “薇娅的女人骑士们” (*Viya’s women and knights*, 4), “盆友们” (non-standard and deviant form of *friends*, 1), “时尚达人们” (*fashion icons*, 1), “体验官们” (*experience officers*, 8). These terms help to either construct intimacy between herself and her followers or show respect to her followers.

Although Li also uses some internet buzzwords to address his followers, he also uses some intimate addressing terms to address his followers by their genders, such as “(所有)女生” (*all girls*, 22), “(所有)女生男生” (*all girls and boys*, 4), “MM们”(*pinyin* acronym of *beauties*, 4), “美眉们” (*beauties*, 1), “宝妈宝爸们” (*moms and daddies*, 3). This further demonstrates that Li prefers to use gender as a specific strategy in his promotion.

## Discussion and conclusion

An analysis of the posts of the three top KOLs at three different levels finds that they tend to construct different images. Luo tends to construct a “*zhinan*” (straight male) image. He prefers to highlight the masculine traits of “forceful” and “rational,” the speech acts of “directive” and “showing judgement and appreciation,” and the use of positive self-addressing terms and negative other-addressing terms. Li tends to construct a “*guimi*” (ladybro) image. He prefers to underline the feminine personality traits of “caring” and “congenial,” the speech acts of “self-reporting moment and information” and “eliciting response,” and the self-denigration self-addressing terms and respectful other-addressing terms. Viya tends to construct a “professional” image. She prefers to underscore both the masculine traits of “forceful” and “confident” and the feminine trait of “understanding,” the speech acts of “promoting self and others” and “self-reporting mood,” and the respectful self- and other-addressing terms.

Their different ways of identity constructions can be attributed to their target consumers and the products they promote. Since the majority of Luo’s followers are males, they support him because of his sharp words and entrepreneurial spirits. Therefore, Luo prefers to construct himself as a “*zhinan*” who is rational, critical, and brave enough to show his judgment towards some social phenomena. In other words, he attracts his followers by distinguishing himself from his followers. By contrast, since the products Li promotes are primarily female products and most of his followers are female. He has to cater to the interest of his followers by constructing himself as a congenial and caring “*guimi*” (ladybro) of his followers. In order to establish solidarity with female followers, he has to resort to “self-reporting moment and information” and “eliciting response” to attract his followers. In between is Viya. Although she is a female, she promotes products to both males and females. She attracts her followers by her expertise in selecting high-quality products with the lowest prices. Therefore, gender does not serve as a prominent promoting strategy because she sells products to both males and females.

Therefore, different KOLs tend to construct different images on their social media. Although previous studies show that feminine traits are gaining advantage over time (Yarchi and Samuel-Azran, 2018; Eagly et al., 2020) as it can “textually harness affect” (Thelwall et al., 2010, p. 1), this study demonstrates that the relations between gender and interaction cannot be taken for granted, because both masculine and feminine traits can be employed to attract different groups of followers (Cooper, 2009; Baxter, 2011; Cameron and Shaw, 2016). The choice of different personality traits and strategies tends to vary with the products they sell and the genders of their followers (Li, 2019). In this study, gender serves as a prominent factor in Luo’s and Li’s performances on social media rather than Viya’s. While playing the gender card can be an effective means to attracting followers of different genders (Craig et al., 2021), it also runs the risk of confining the promotion to a certain group (Sandel and Wang, 2022), thus failing to expand the influences across genders. That may explain why Viya, without playing the gender card, ranks as the most influential KOL among the three, because she relies on her expertise rather than her gender to promote her products.

Social media provides a platform for people to construct distinguishing personalities (Sandel and Wang, 2022). Although gender resource is frequently employed for fan-based interactions (Zhang and Hjorth, 2019), it has been relatively overlooked (Zhang and Hjorth, 2019) in contrast to the fast-growing live-streaming e-commerce in China.

This study reveals not only the different strategies by different top KOLs to interact with their followers on social media but, more importantly, the dynamic relations between gender and interaction. It demonstrates that gender serves as not only a social constraint but also an important social resource which can be appropriated and explored for branding on social media (Duffy and Hund, 2015). Therefore, instead of examining whether gender matters online or not, it is more meaningful and significant to examine how gender is explored strategically by different users on social media to serve the intended communicative purposes (Rellstab, 2007). A combination of the methods and theories in critical discourse analysis, gender, and pragmatics in this study has demonstrated the potentials of CDA in explicating the dialectic and complex relations between gender and interaction on social media (Chang et al., 2016). Nevertheless, this study only addresses the three top KOLs, and future studies will examine to what extent gender serves as a prominent promotion strategy by those influential KOLs in China and other countries. Besides, the communication effects of different gender-related strategies also merit our further attention. It is expected that they can generate more illuminating findings on the relations between gender and interaction by top KOLs on social media.

## Data availability statement

The raw data supporting the conclusions of this article will be made available by the authors, without undue reservation.

## Author contributions

ML contributed to the research design, data description and interpretation, paper writing and revising, and funding support. RZ analyzed the data, and contributed to the literature review and paper drafting. JF was responsible for the quality control and proofreading. All authors contributed to the article and approved the submitted version.

## Funding

This work was funded by the Hong Kong Polytechnic University (UGC; Project ID: 1-BE68).

## Conflict of interest

The authors declare that the research was conducted in the absence of any commercial or financial relationships that could be construed as a potential conflict of interest.

## Publisher’s note

All claims expressed in this article are solely those of the authors and do not necessarily represent those of their affiliated organizations, or those of the publisher, the editors and the reviewers. Any product that may be evaluated in this article, or claim that may be made by its manufacturer, is not guaranteed or endorsed by the publisher.
